# Supplementary material for: Metavalently bonded tellurides: the essence of improved thermoelectric performance in elemental Te
Source: Nat Commun. 2024 Apr 12;15:3177. doi: 10.1038/s41467-024-47578-w (PMC11014947; doi:10.1038/s41467-024-47578-w)
Supplement: Supplementary file 1 — Supplementary Information [file 41467_2024_47578_MOESM1_ESM.pdf]

Supplementary Information for

Metavalently bonded tellurides: the essence of improved thermoelectric performance in elemental Te

Decheng An[1], Senhao Zhang[2], Xin Zhai[3], Wutao Yang[1], Riga Wu[2], Huaide Zhang[2], Wenhao Fan[4], Wenxian Wang[4], Shaoping Chen[4], Oana Cojocaru-Mirédin[5], Xian-Ming Zhang[1,4*], Matthias Wuttig[2,6*], and Yuan Yu[2*]

[1] College of Chemistry, Taiyuan University of Technology, Taiyuan 030024, China

[2] Institute of Physics (IA), RWTH Aachen University, Sommerfeldstraße 14, 52074 Aachen, Germany

[3] School of Electronic Science & Engineering, Southeast University, Nanjing 210096, China

[4] Key Laboratory of Interface Science and Engineering in Advanced Materials, College of Materials Science and Engineering, Instrumental Analysis Center, Taiyuan University of Technology, Taiyuan 030024, China

[5] Department of Sustainable Systems Engineering (INATECH), Albert-Ludwigs-Universität Freiburg, 79110 Freiburg, Germany

[6] Peter Grünberg Institute (PGI 10), Forschungszentrum Jülich, 52428 Jülich, Germany

[*]Corresponding author: zhangxianming@tyut.edu.cn (X.-M. Z.); wuttig@physik.rwth-aachen.de (M. W.); yu@physik.rwth-aachen.de (Y. Y.)

Supplementary Figures

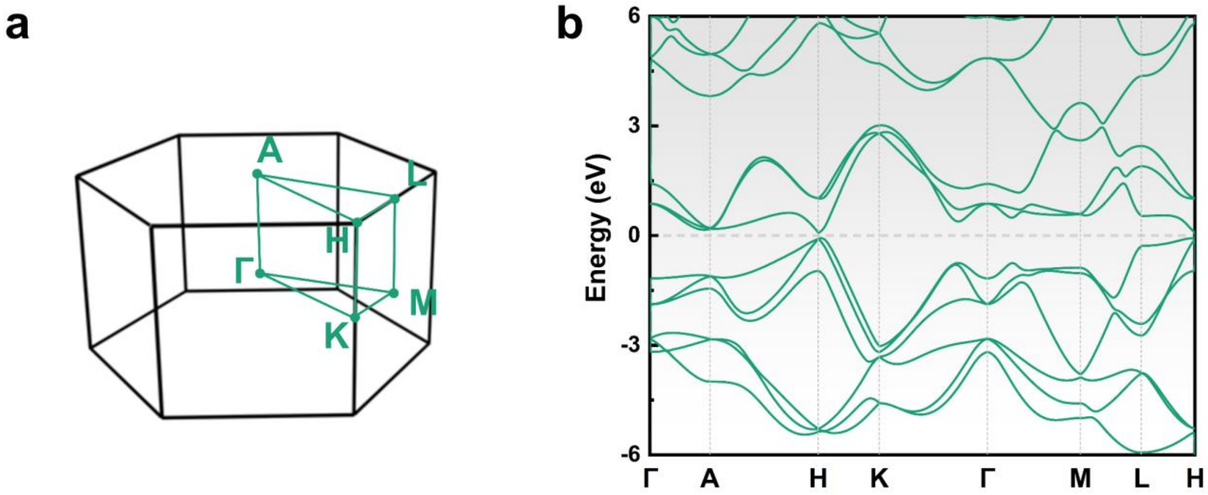

Supplementary Fig. 1. First-principle calculation of electronic band structure. a Bulk Brillouin zone of trigonal Te. **b** Calculated band structure for Te.

In **Supplementary Fig. 1**, bulk Te is found to be a direct semiconductor with a band gap of $E_g = 0.19$ eV. In fact, this theoretical band gap is underestimated in comparison to the reported experimental value of ~ 0.3 eV[1]. The inherently low carrier concentration (~ 1×10^{17} cm^{-3}) is mainly responsible for the poor thermoelectric performance in pristine Te, whereas excellent thermoelectrics are normally heavily doped semiconductors[2] with a carrier concentration between 10^{19}–10^{21} cm^{-3}. Generally, for compounds containing heavy atoms, spin-orbit coupling (SOC) influences the electrical properties such as the band gap value and the spin splitting effect[3, 4]. However, it has been shown by Han et al. that the bandgap of Te will be significantly underestimated if the SOC effect is included in the calculation[5]. Thus, the electronic band structure of elemental Te as shown in **Supplementary Fig. 1** does not include the SOC effect. Similar treatments and results have also been reported elsewhere[6].

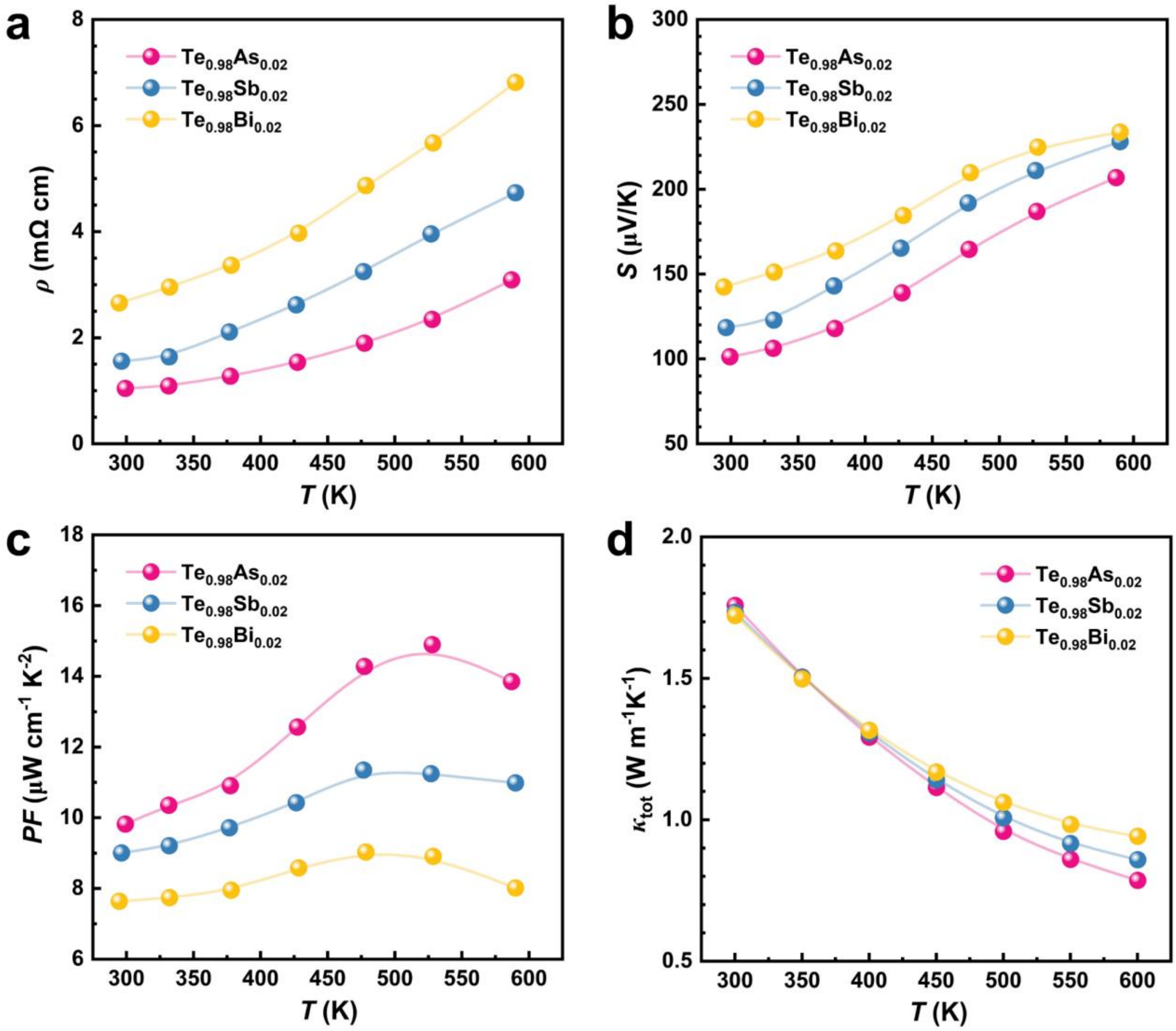

Supplementary Fig. 2. Temperature-dependent thermoelectric transport properties of $Te_{0.98}As_{0.02}$, $Te_{0.98}Sb_{0.02}$, and $Te_{0.98}Bi_{0.02}$. a Electrical resistivity (ρ). **b** Seebeck coefficient (S). **c** Power factor (PF = S^2/ρ). **d** Total thermal conductivity (κ_{tot}). For simplifying the following studies, we set the doping content (x%) uniformly at 2% in the series of $Te_{1-x}M_x$ (M = As, Sb, and Bi) samples, and thus the corresponding thermoelectric performances have not been adequately optimized in this work in view of their different optimum doping level[1, 7, 8].

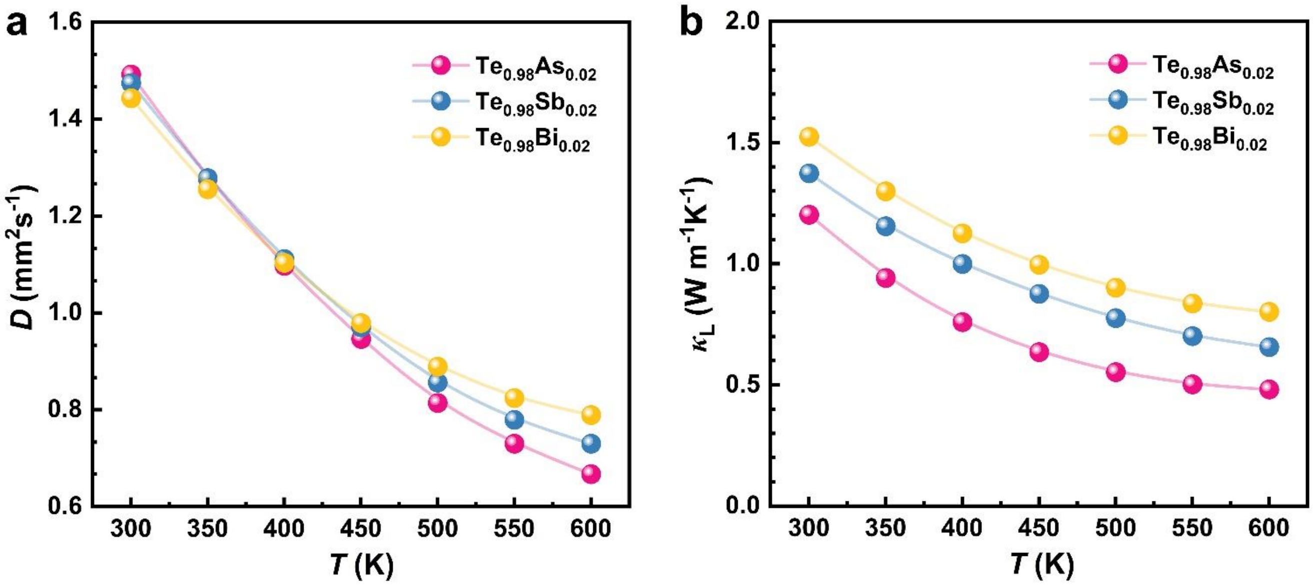

Supplementary Fig. 3. Temperature dependences of thermal properties of $Te_{0.98}As_{0.02}$, $Te_{0.98}Sb_{0.02}$, and $Te_{0.98}Bi_{0.02}$. **a** Thermal diffusivity (*D*). **b** Lattice thermal conductivity (κ_L).

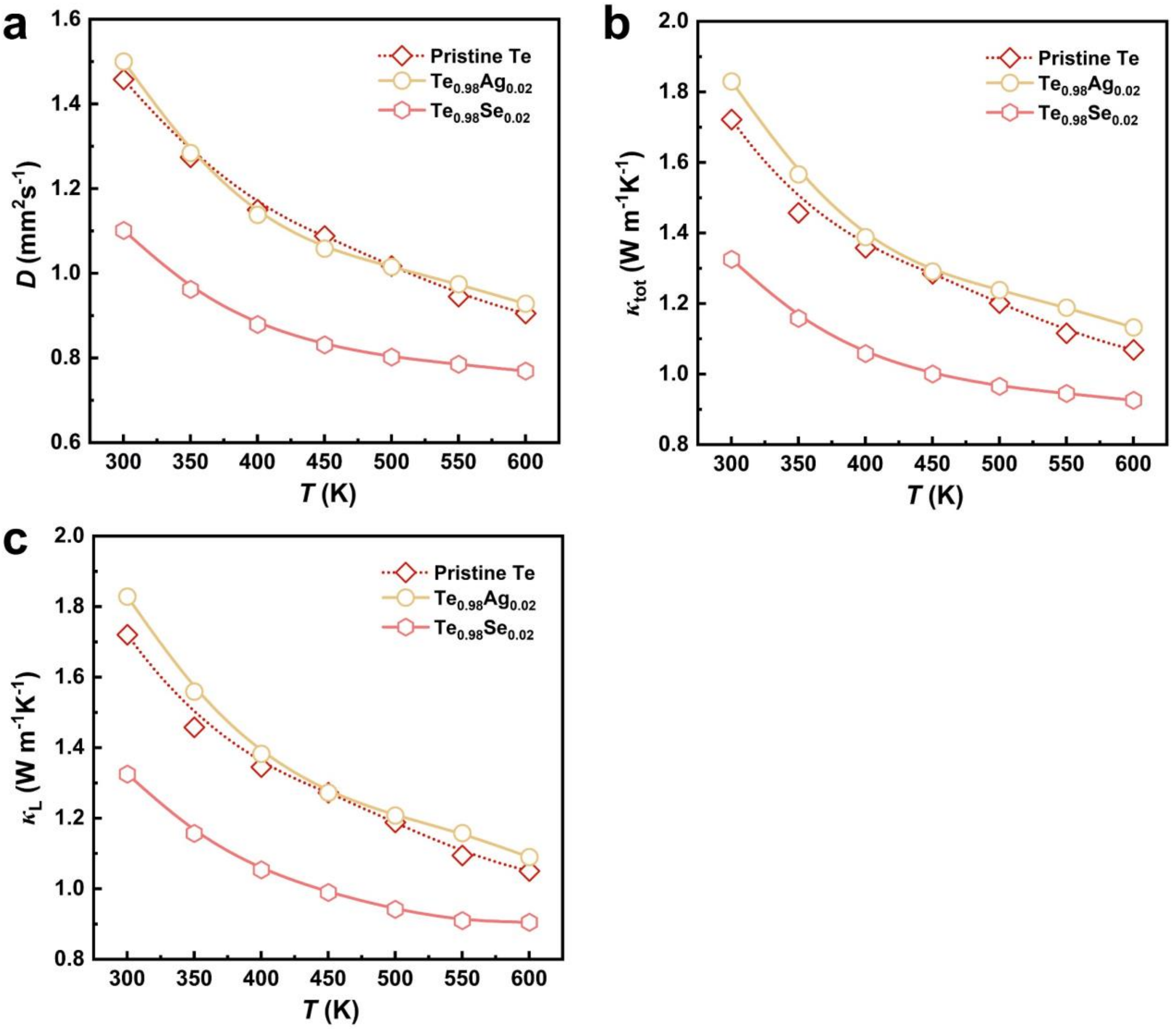

Supplementary Fig. 4. Temperature dependences of thermal properties of pristine Te, $Te_{0.98}Ag_{0.02}$, and $Te_{0.98}Se_{0.02}$. a Thermal diffusivity (D). **b** Total thermal conductivity (κ_{tot}). **c** Lattice thermal conductivity (κ_L).

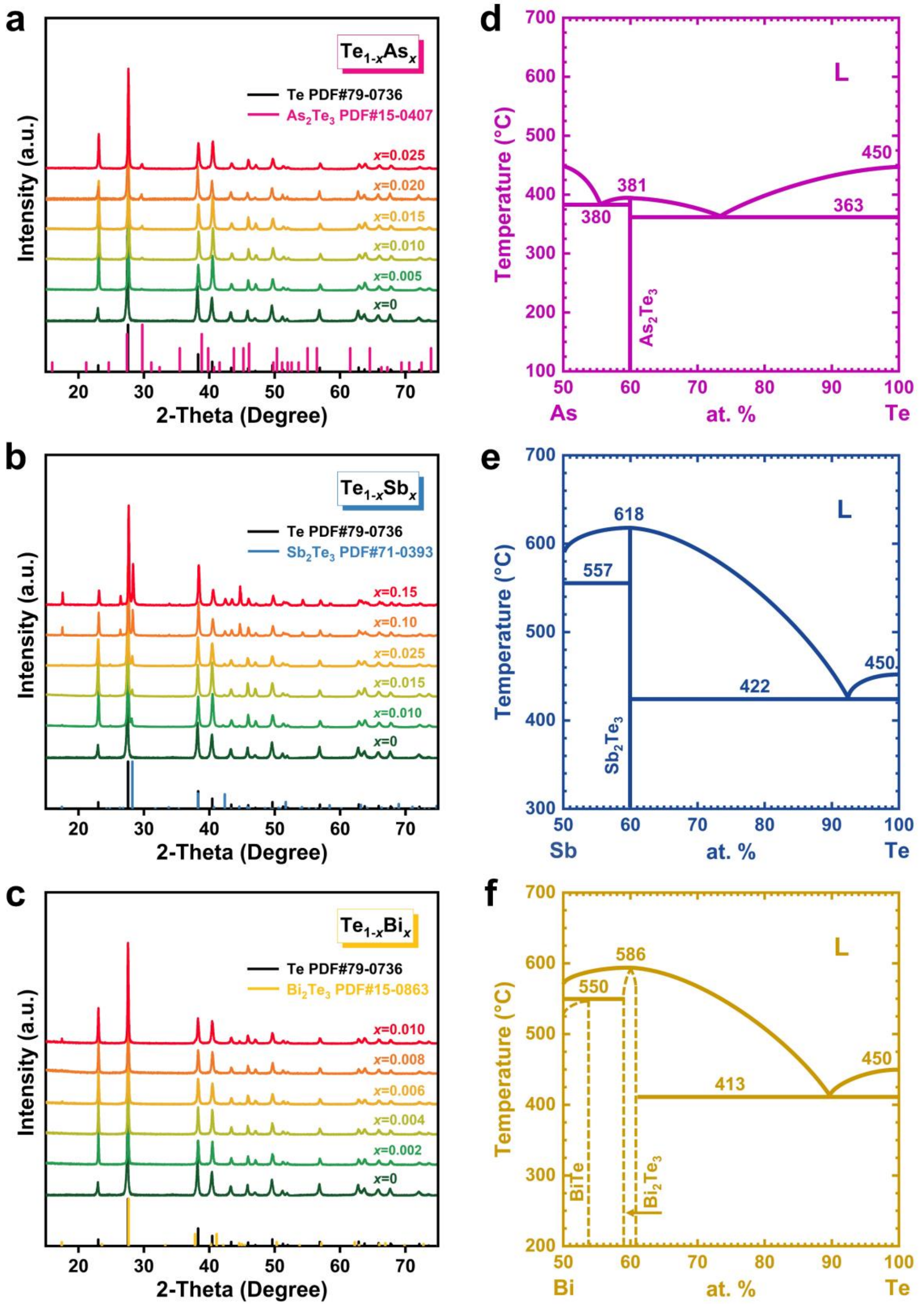

Supplementary Fig. 5. Phase compositions of doped Te systems. Room-temperature powder XRD patterns of **a** $Te_{1-x}As_x$ (x = 0.005−0.025), **b** $Te_{1-x}Sb_x$ (x = 0.0−0.15), and **c** $Te_{1-x}Bi_x$ (x = 0.002−0.01) samples. Binary phase diagrams for the **d** As-Te, **e** Sb-Te, and **f** Bi-Te systems.

In **Supplementary Fig. 5a-c**, it is obvious that the intensity of the diffraction peak of telluride secondary phase increases gradually with increasing x content in all three series of samples.

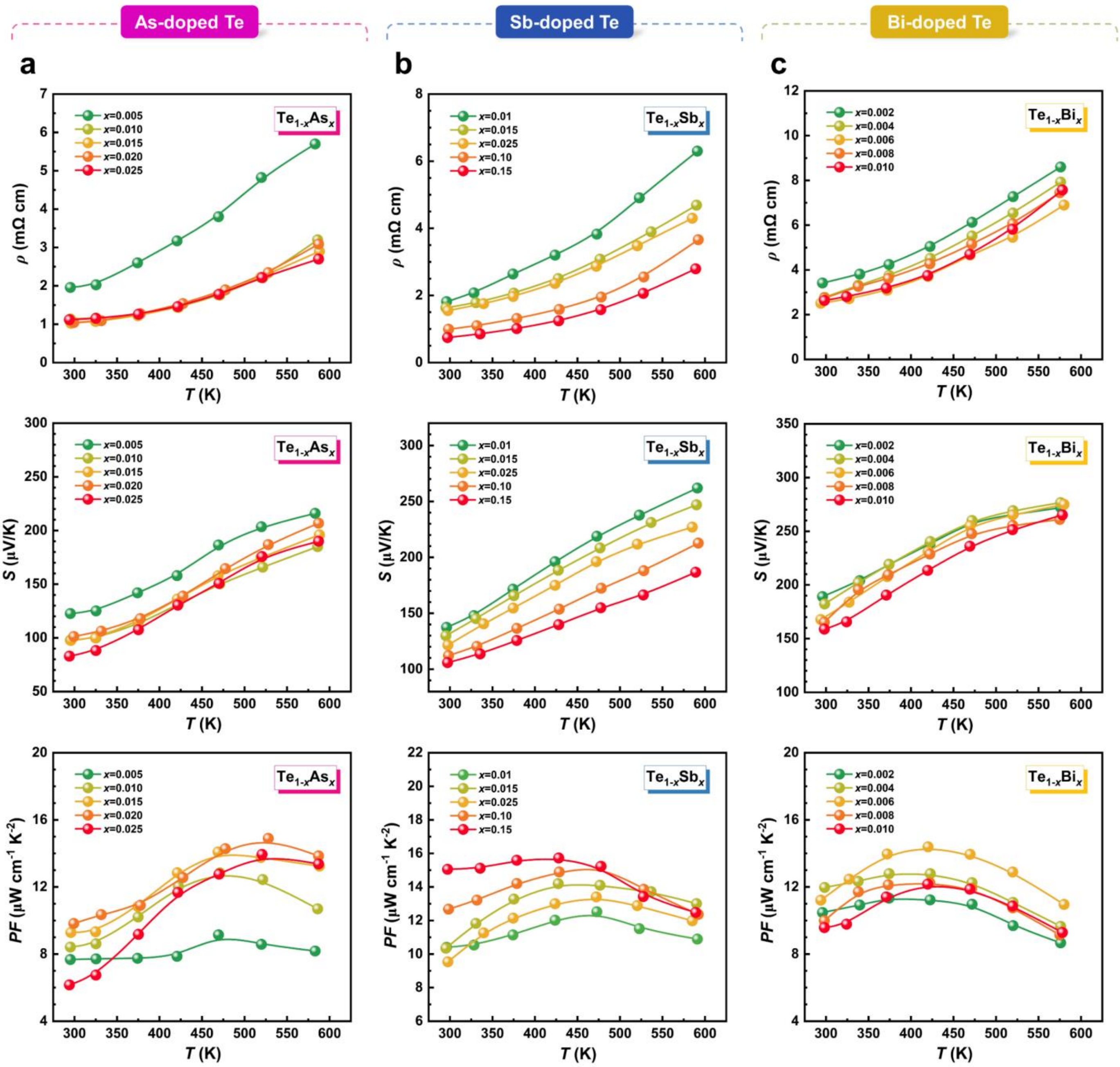

Supplementary Fig. 6. Group-VA-elements (As, Sb, and Bi) induced electrical property modulation. Temperature-dependent electrical transport properties including electrical resistivity (ρ), Seebeck coefficient (S) and power factor (PF = S^2/ρ) for bulk **a** $Te_{1-x}As_x$ (x = 0.005−0.025), **b** $Te_{1-x}Sb_x$ (x = 0.01−0.15), and **c** $Te_{1-x}Bi_x$ (x = 0.002−0.01) samples.

In **Supplementary Fig. 6**, as a result of the remarkable reduction of electrical resistivity through As/Sb/Bi doping, the PFs of the doped bulk samples are improved by an order of magnitude in comparison with those of the pristine Te[9]. It is worth noting that the maximum of PF respectively reaches up to 14.9, 15.7, and 14.4 μW cm^{-1} K^{-2} in $Te_{0.98}As_{0.02}$ (Fig. 6a; bottom), $Te_{0.85}Sb_{0.15}$ (Fig. 6b; bottom), and $Te_{0.994}Bi_{0.006}$ (Fig. 6c; bottom), even though all beyond their separate solubilities. In addition, in all samples, both the ρ and S monotonically increase with elevating temperature, demonstrating a **degenerate semiconducting behavior**.

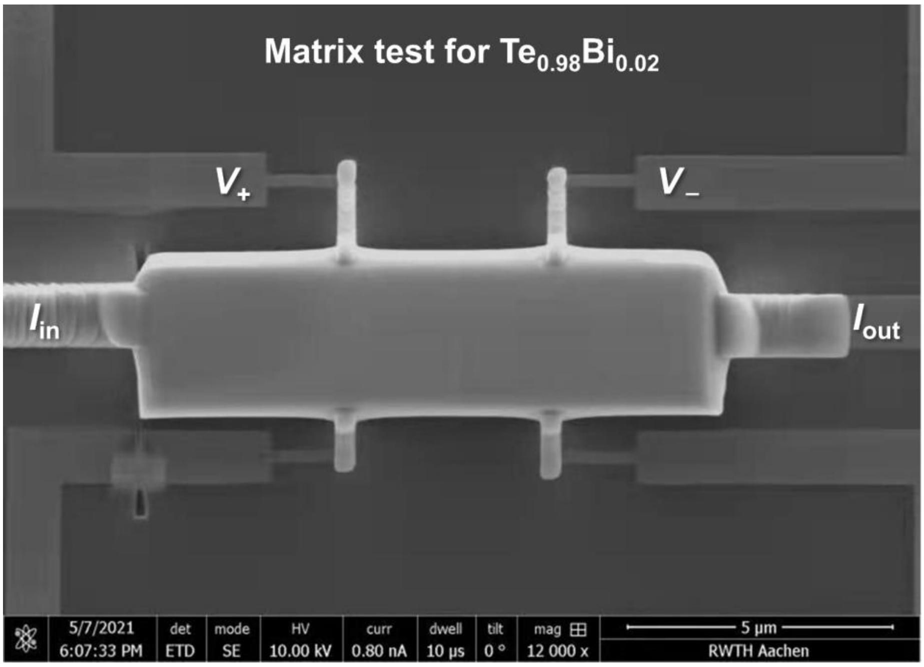

Supplementary Fig. 7. Local transport property measurement platform. A scanning electron microscope image of a Hall-bar geometry device for the measurement of the electrical conductivity in a nominal $Te_{0.98}Bi_{0.02}$ sample that does not contain precipitate (namely its matrix portion). The measured sample is a cuboid of around 1.5 µm by 3 µm by 10 µm.

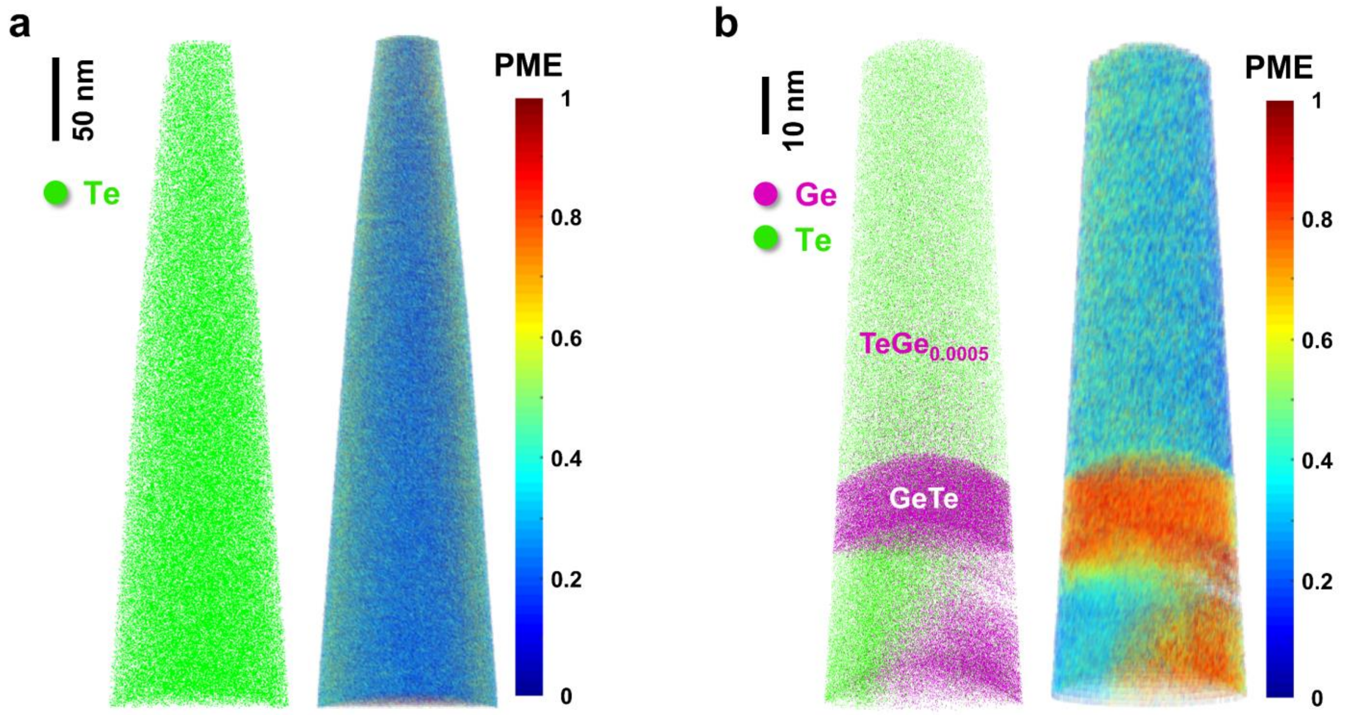

Supplementary Fig. 8. Probability of multiple events (PME) maps. a Pure Te sample. **b** Ge-doped Te with a nominal composition $Te_{0.98}Ge_{0.02}$.

The multiple events described the correlated field evaporation process of more than one ion dislodged by a successful laser pulse (an unsuccessful laser pulse cannot produce any detected ion dislodgement). It has been proven in many studies that the large PME value (>50%) is prevailing characteristic for all materials employing "metavalent" bonding (MVB), whereas the value is often lower than 30% for non-MVB materials[10]. In **Supplementary Fig. 8**, a low PME of <30% is found for both the pure Te and the matrix region of Ge-doped Te, and thus is characteristic for the other bonding mechanisms that is, ionic or covalent bonding. In contrast, the rhombohedral GeTe precipitates show a high probability of multiple events of more than 70%, and is indicative of MVB. We have recently suggested that MVB are characterized by roughly one electron being shared between nearest neighbor atoms and a modest long-range electron transfer. That is to say, their bonding orbitals mixed with the atomic environment beyond the nearest neighbor atoms but not as far as in metallic materials. Even upon breaking a separate bond, the second-nearest neighbor bonding interactions were intensive enough such that multiple events are witnessed. Note that the solubility of Ge is not more than 0.05% in Te based on the APT characterization.

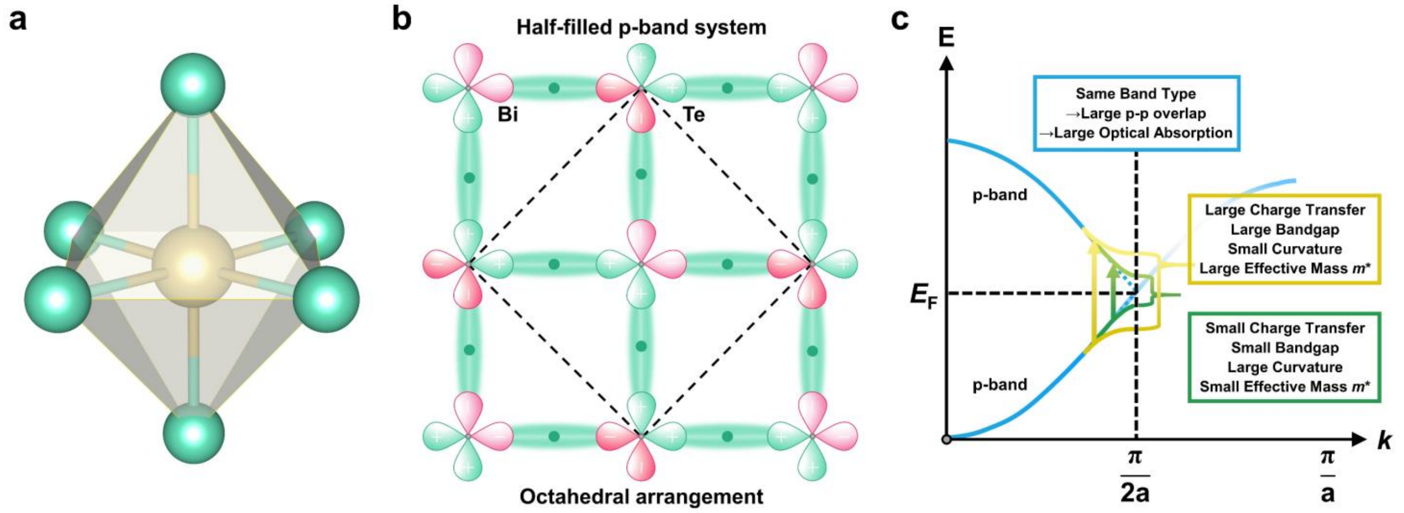

Supplementary Fig. 9. Metavalently bonded Bi_2Te_3. a Octahedral-like coordination in Bi_2Te_3. **b** A 2D octahedral atomic arrangement of Bi_2Te_3. Atomic orbitals of Bi and Te responsible for bond formation in Bi_2Te_3. σ-bonds are formed from p-orbitals, which are occupied by about half an electron pair, resulting in a metallic ground state. **c** The resulting band structure.

As seen in **Supplementary Fig. 9**, an octahedral atomic arrangement can be observed in rocksalt-type Bi_2Te_3. Since there is very little s-p hybridization[11, 12], this arrangement is a result of the σ-bonds formed between adjacent atoms because of the half-filled band of p-electrons (i.e., on average three p-electrons per site). Here we include one vacancy per quintuple layer. Energy minimization can be realized by the formation of σ-bonds in Bi_2Te_3. Nonetheless, this electronic configuration remains unstable since the electronic energy can either be reduced by a structural distortion or by charge transfer between adjacent atoms[13]. In Bi_2Te_3, a small charge transfer from Bi to Te and significant electron sharing (moderate Peierls distortion) would result in an almost metallic band[14], the overlap of adjacent p-orbitals of Bi and Te atoms dominates the valence and conduction band. The larger the orbital overlap, the smaller the corresponding bandgap, which causes a large curvature of the bands in the vicinity of the zone boundary (L-point of the Brillouin zone). In this case, the resulting band structure shows a small band effective mass m_b^*, a high valley degeneracy N_V, and a high band anisotropy K[15, 16]. This in turn enhances the Fermi surface complexity factor ($N_V^*K^*$) that describes the ability of different energy bands to maximize the power factor[17]. This unconventional bonding mechanism prevails in β-As_2Te_3 and Sb_2Te_3[18, 19].

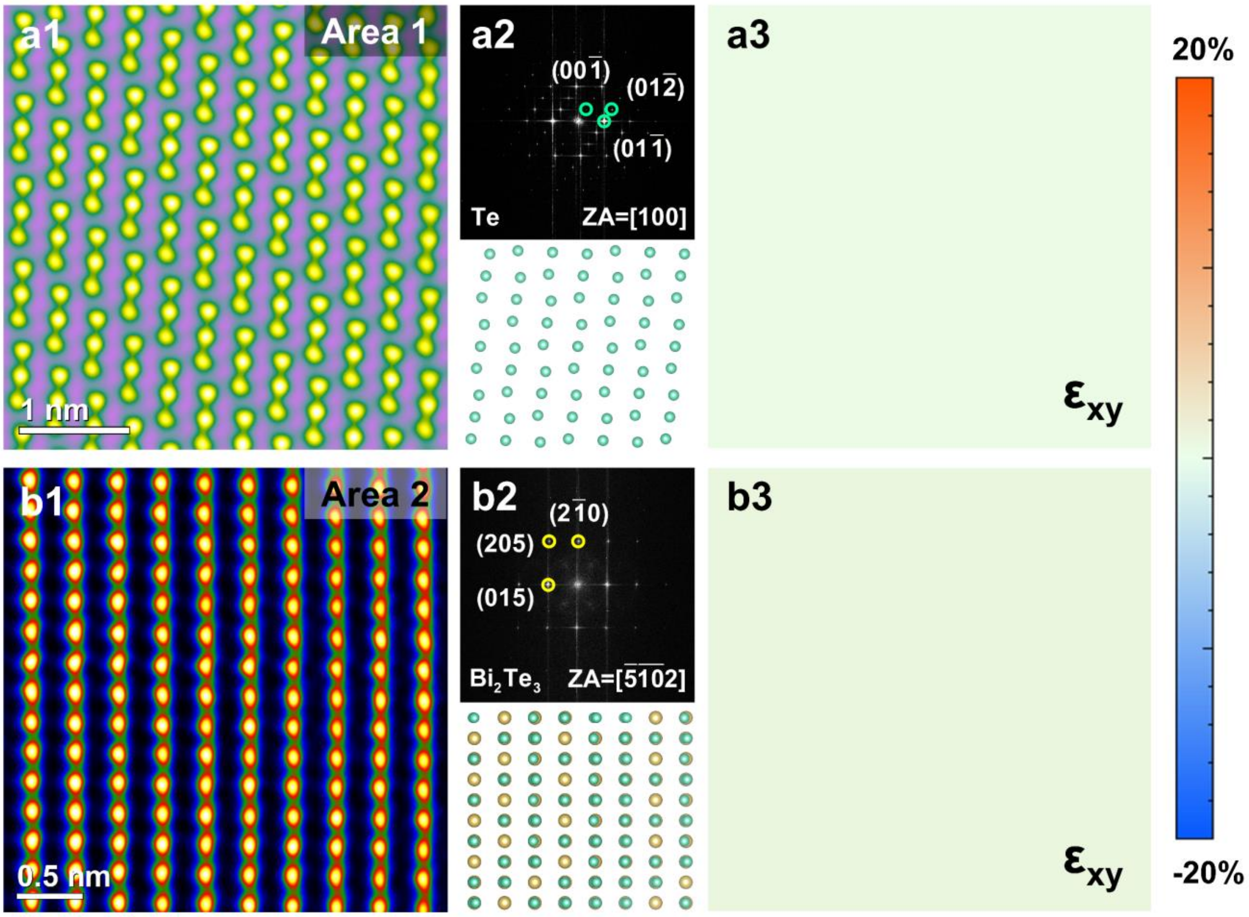

Supplementary Fig. 10. Visible observation from atomic-level STEM. Magnified HAADF-STEM images of **Fig. 5b** and **5c**, showing the atomic arrangements for **a1** Te and **b1** Bi_2Te_3, respectively. FFT patterns and crystal model structures for **a2** Te viewed along [100] direction and **b2** Bi_2Te_3 viewed along [$\overline{5}\overline{1}02$] direction. Corresponding geometric phase analysis (GPA) of the areas of **a1** and **b1**, confirming the inexistence of visible lattice strain for both **a3** Te and **b3** Bi_2Te_3.

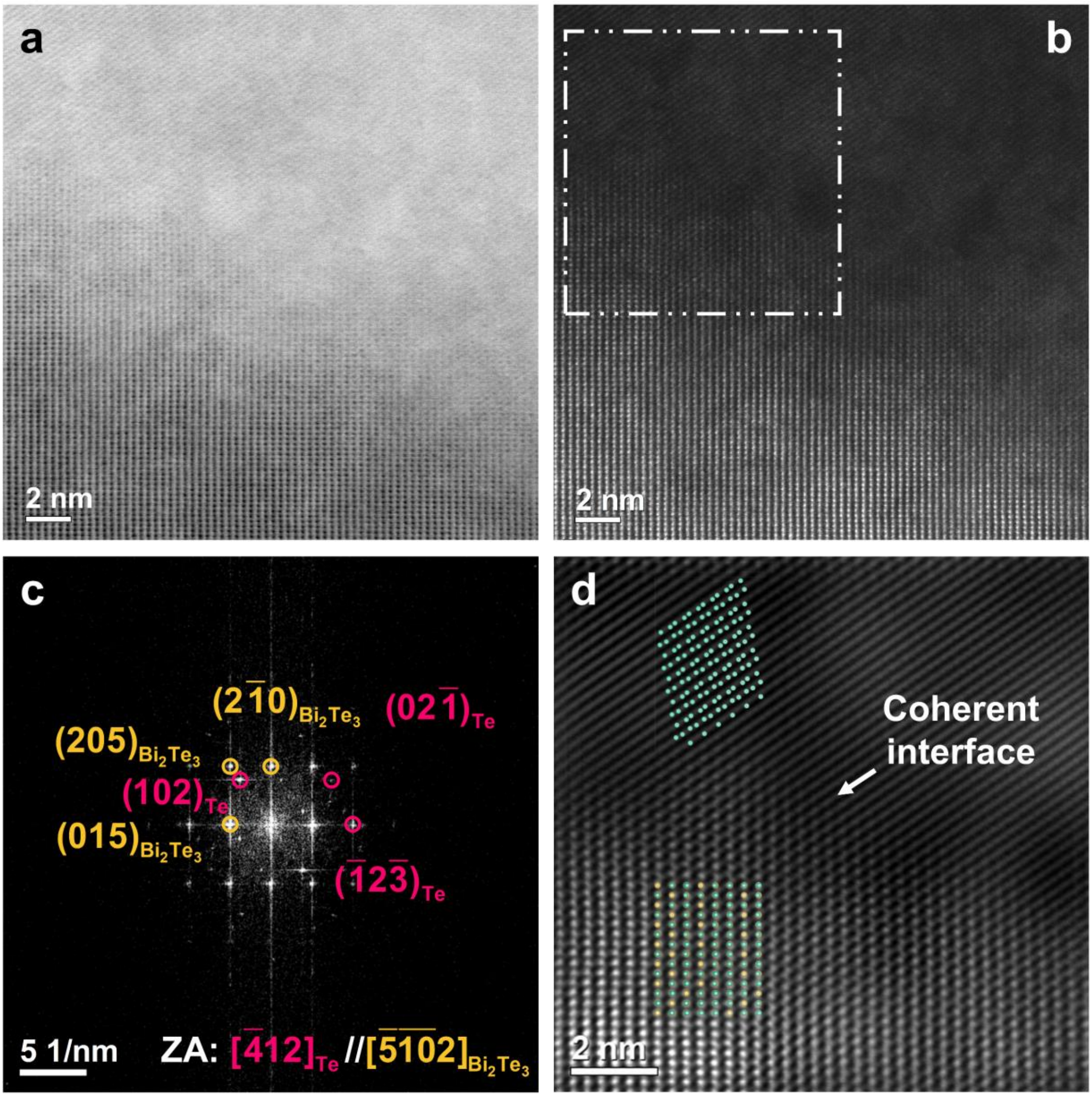

Supplementary Fig. 11. Structure of Bi_2Te_3/Te interface. Atomic-scale **a** ABF-STEM and **b** HAADF-STEM images displaying the interface between the Bi_2Te_3 and the Te matrix. **c** FFT and corresponding **d** inverse FFT at the interface (corresponding to the area marked by the dashed white square in **b**), showing a coherent interface. The Te (green) and Bi (yellow) atoms are overlayed on the image.

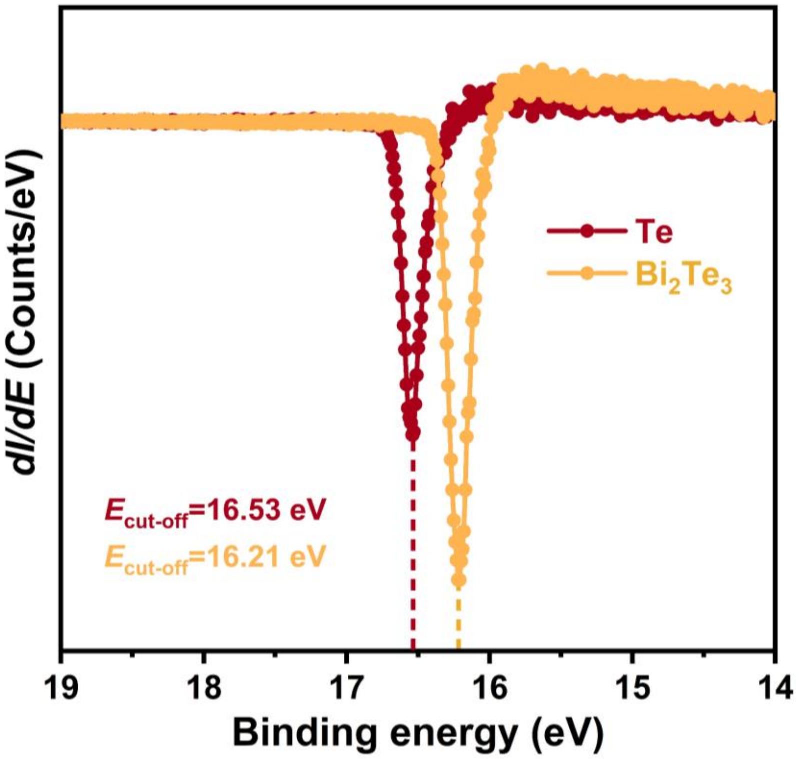

Supplementary Fig. 12. Ultraviolet photoelectron spectroscopy (UPS) plots. The binding energy differential of the cut-off edge intensity for Te and Bi_2Te_3. The E_{cutoff} of 16.53 eV for Te and 16.21 eV for Bi_2Te_3 could be derived by differentiating the cut-off edge.

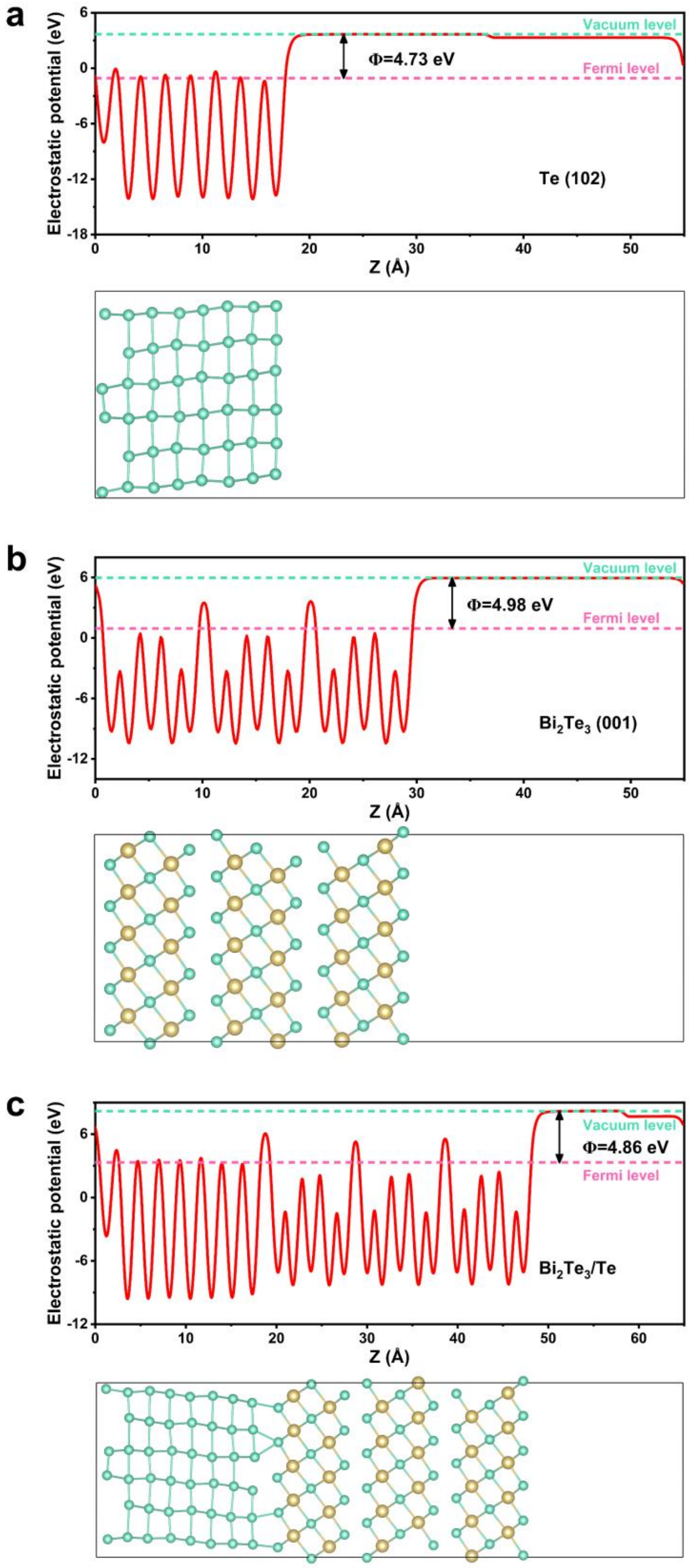

Supplementary Fig. 13. Work function from DFT calculations. Electrostatic potentials of **a** Te, **b** Bi_2Te_3, and **c** the Bi_2Te_3/Te heterostructure along the Z direction (pointing from Te to Bi_2Te_3). Green and yellow spheres stand for Te and Bi atoms, respectively. Dashed green and pink lines represent the vacuum and Fermi energy levels.

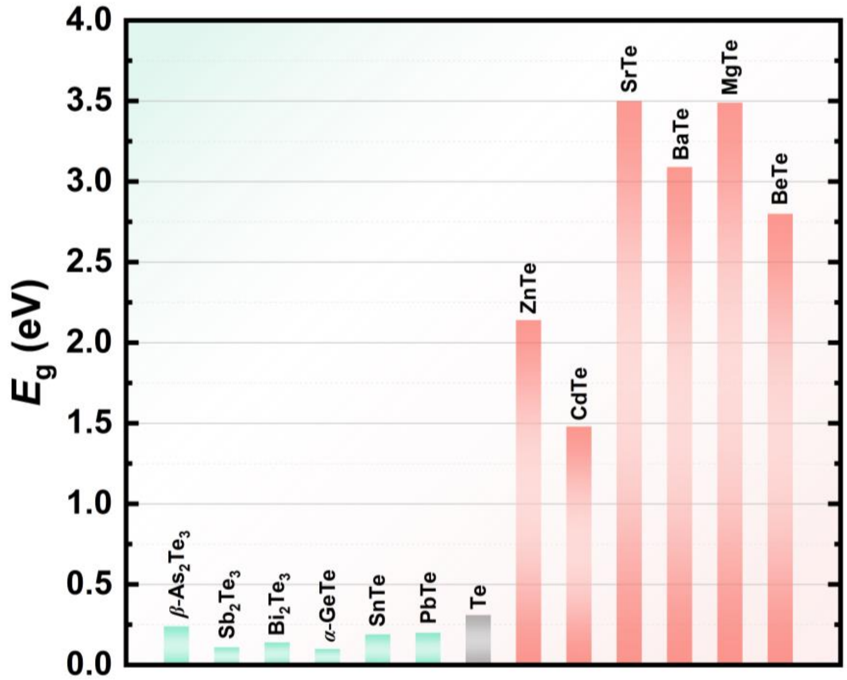

Supplementary Fig. 14. Bandgaps for undoped *p*-type tellurides. These tellurides can be divided into two groups regarding their bandgaps[16, 20, 21, 22, 23, 24]: tellurides with E_g values larger than 0.31 eV are displayed in red, while those with E_g below 0.31 eV are depicted in green. The E_g of Te is 0.31 eV.

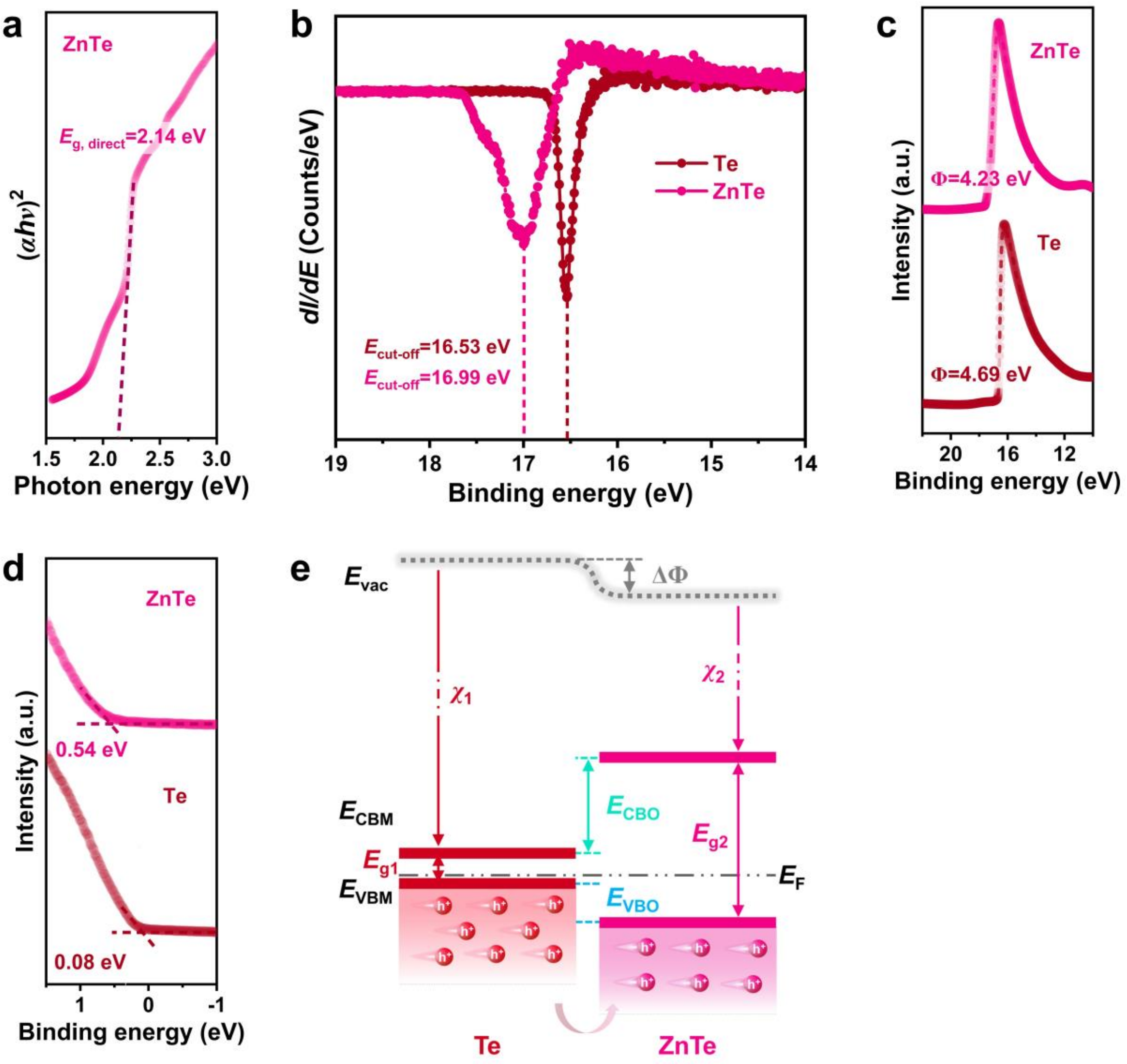

Supplementary Fig. 15. Interfacial characteristics of the *p*-type Te/ZnTe heterostructure. a The band gap of covalent bonded ZnTe. A Tauc plot of the optical-absorption coefficient, α, that is $(\alpha h\nu)^2$, versus photon energy, $h\nu$, of ZnTe shows a bandgap of 2.14 eV. **b, c** The work functions, Φ, of 4.23 eV for ZnTe and 4.69 eV for Te are evaluated by ultraviolet photoelectron spectroscopy (UPS) plots. **d** The valence band maximum (VBM) positions for ZnTe of 0.54 eV and Te of 0.08 eV. **e** The experimentally determined electronic band diagrams of ZnTe and Te, indicating the presence of energy-filtering effect[25, 26] at the Te/ZnTe interface (with a 0.46 eV barrier height). Indeed, such a large positive E_{VBO} is outside the tolerable range (within −0.4 to +0.3 eV) of valence band alignments.

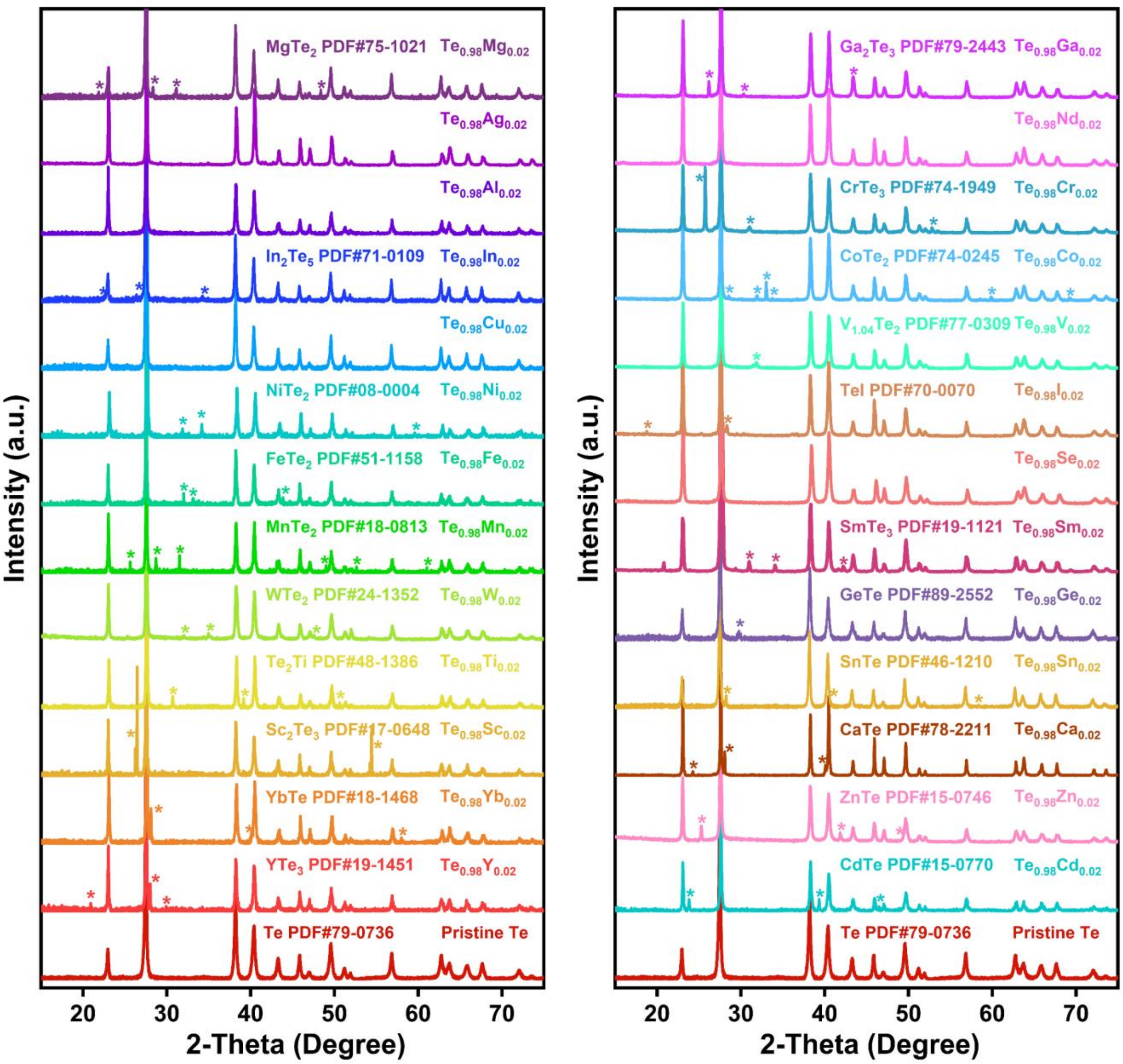

Supplementary Fig. 16. Analysis of the chemical composition for doped Te materials. XRD patterns at room temperature for 26 different $Te_{0.98}M_{0.02}$ samples and pristine Te, revealing the dual-phase structure consisted of a trigonal Te matrix and corresponding tellurides, except for the individual case showing the single-phase structure because of the X-ray diffraction detection limits.

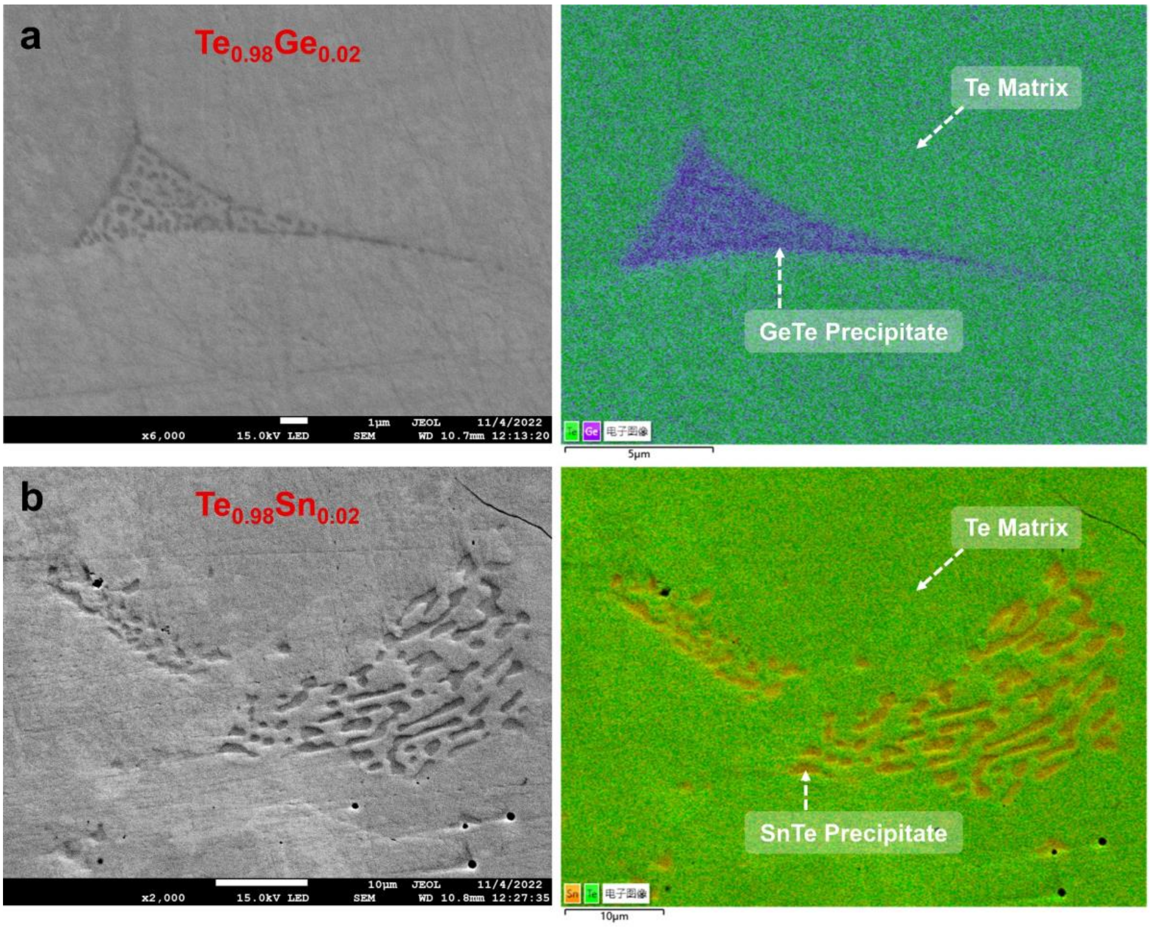

Supplementary Fig. 17. Microscopic analysis of precipitate morphology. SEM images and the corresponding EDS composition mappings of **a** $Te_{0.98}Ge_{0.02}$ and **b** $Te_{0.98}Sn_{0.02}$, showing the visible secondary-phase tellurides distributed in the Te matrix. The compositions of these matrixes and precipitates were determined by the EDS spot analyses (see **Supplementary Table 1**). Note that GeTe and SnTe all employ metavalent bonding as shown in **Fig. 7**.

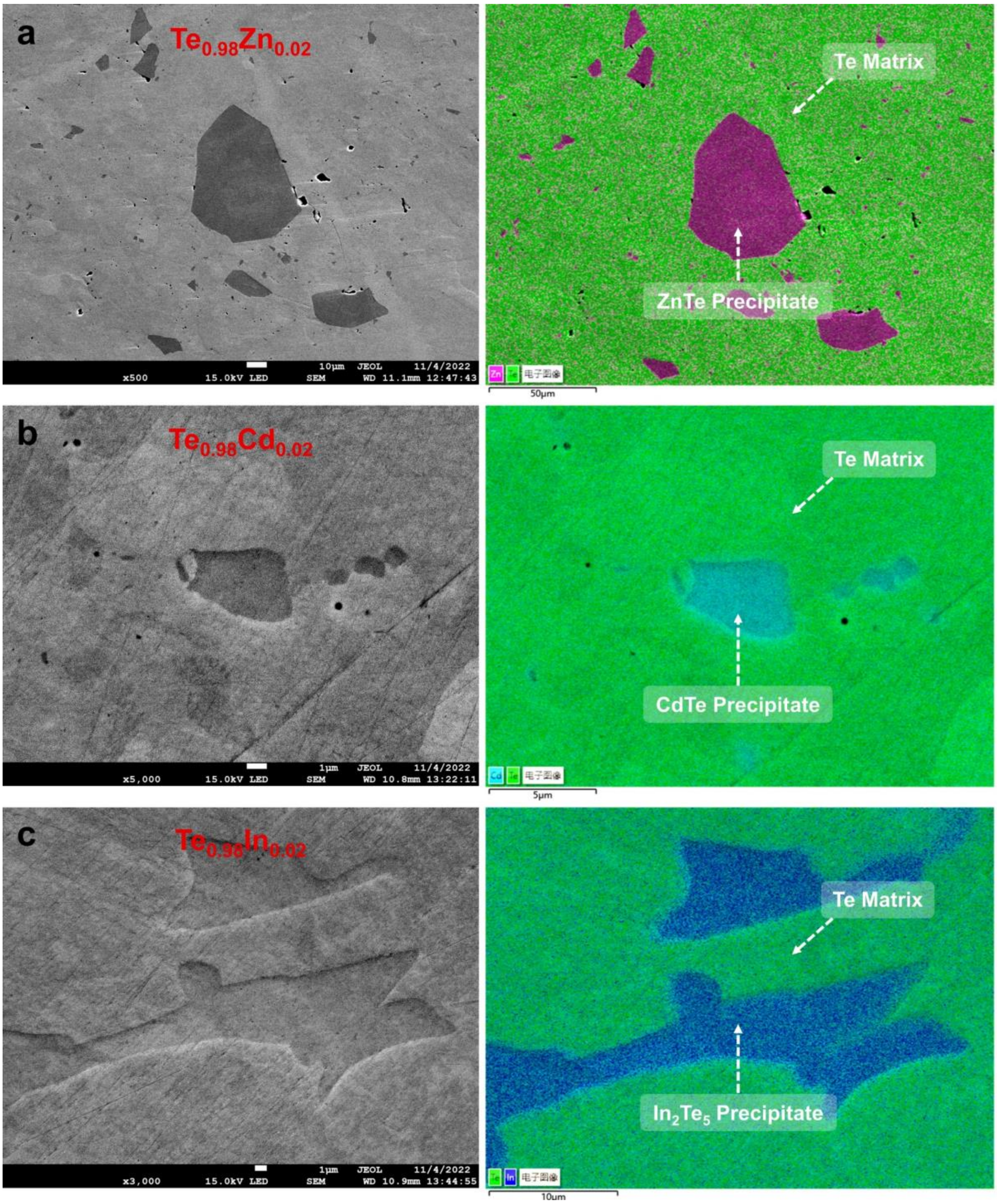

Supplementary Fig. 18. Microscopic analysis of precipitate morphology. SEM images and the EDS mappings for **a** $Te_{0.98}Zn_{0.02}$, **b** $Te_{0.98}Cd_{0.02}$, and **c** $Te_{0.98}In_{0.02}$. The quantitative results of EDS spectrum are listed in **Supplementary Table 1**.

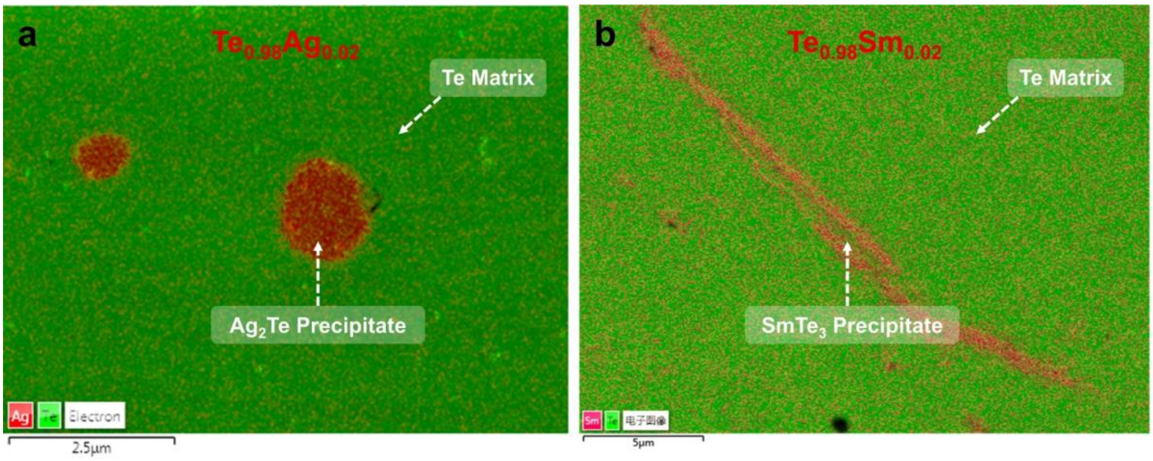

Supplementary Fig. 19. Microscopic analysis of precipitate morphology. EDS mappings for **a** $Te_{0.98}Ag_{0.02}$ and **b** $Te_{0.98}Sm_{0.02}$, where the former have shown the Ag_2Te precipitates that cannot be detected in XRD. The quantitative results of the EDS spectrum are listed in **Supplementary Table 1**.

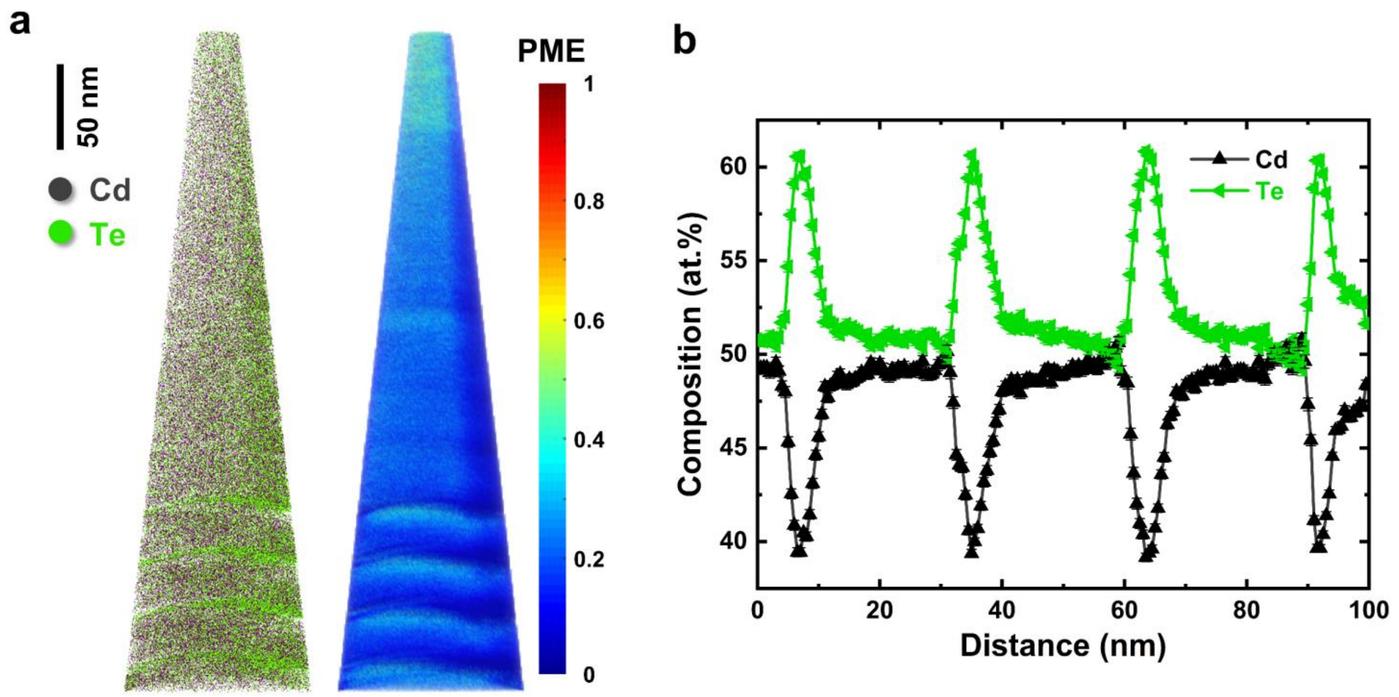

Supplementary Fig. 20. APT analysis results of the CdTe precipitate in $Te_{0.98}Cd_{0.02}$. a 3D APT reconstruction tomogram and the corresponding PME map. Some stacking faults can be observed inside the CdTe precipitate, which can be identified as Cd_2Te_3 by the 1D composition profile shown in **b**.

Supplementary Table 1. EDS spectrum analysis in $Te_{0.98}M_{0.02}$ samples.

Nominal composition	Actual atomic concentration			
	Matrix zone (at.%)		Precipitate zone (at.%)	
**$Te_{0.98}As_{0.02}$**	Te	100	Te	63.9
	As	0	As	36.1
**$Te_{0.98}Sb_{0.02}$**	Te	100	Te	60.1
	Sb	0	Sb	39.9
**$Te_{0.98}Bi_{0.02}$**	Te	100	Te	61.3
	Bi	0	Bi	38.7
**$Te_{0.98}Ge_{0.02}$**	Te	100	Te	50.1
	Ge	0	Ge	49.9
**$Te_{0.98}Sn_{0.02}$**	Te	100	Te	52.5
	Sn	0	Sn	47.5
**$Te_{0.98}Zn_{0.02}$**	Te	100	Te	49.7
	Zn	0	Zn	50.3
**$Te_{0.98}Cd_{0.02}$**	Te	100	Te	49.7
	Cd	0	Cd	50.3
**$Te_{0.98}In_{0.02}$**	Te	100	Te	71.8
	In	0	In	28.2
**$Te_{0.98}Ag_{0.02}$**	Te	100	Te	37.8
	Ag	0	Ag	62.2
**$Te_{0.98}Sm_{0.02}$**	Te	100	Te	76.6
	Sm	0	Sm	23.4

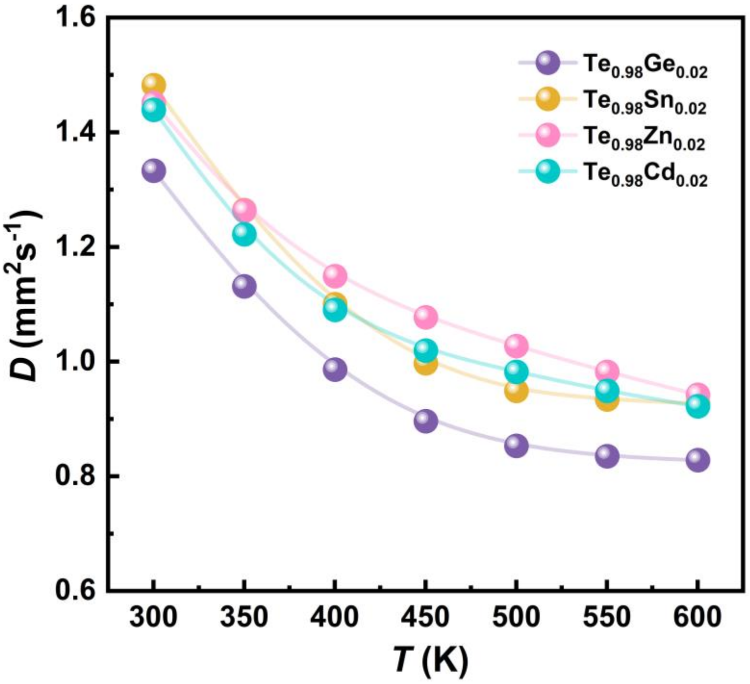

Supplementary Fig. 21. Temperature dependence of thermal diffusivity (*D*) for $Te_{0.98}M_{0.02}$ (M = Ge, Sn, Zn, and Cd) samples.

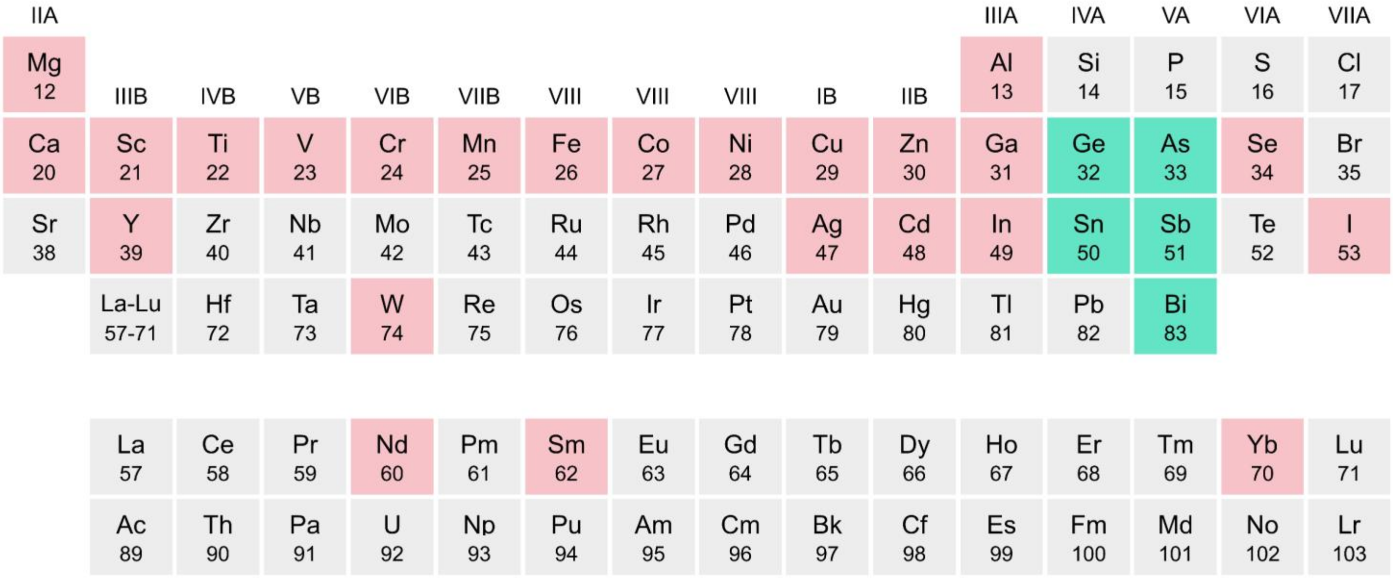

Supplementary Fig. 22. Distribution of elements from the screened dopants. The elements with a green background indicate that their tellurides utilize MVB, whereas the elements with a red background mean that their tellurides employ non-MVB. The gray background color corresponds to the unvalidated elements.

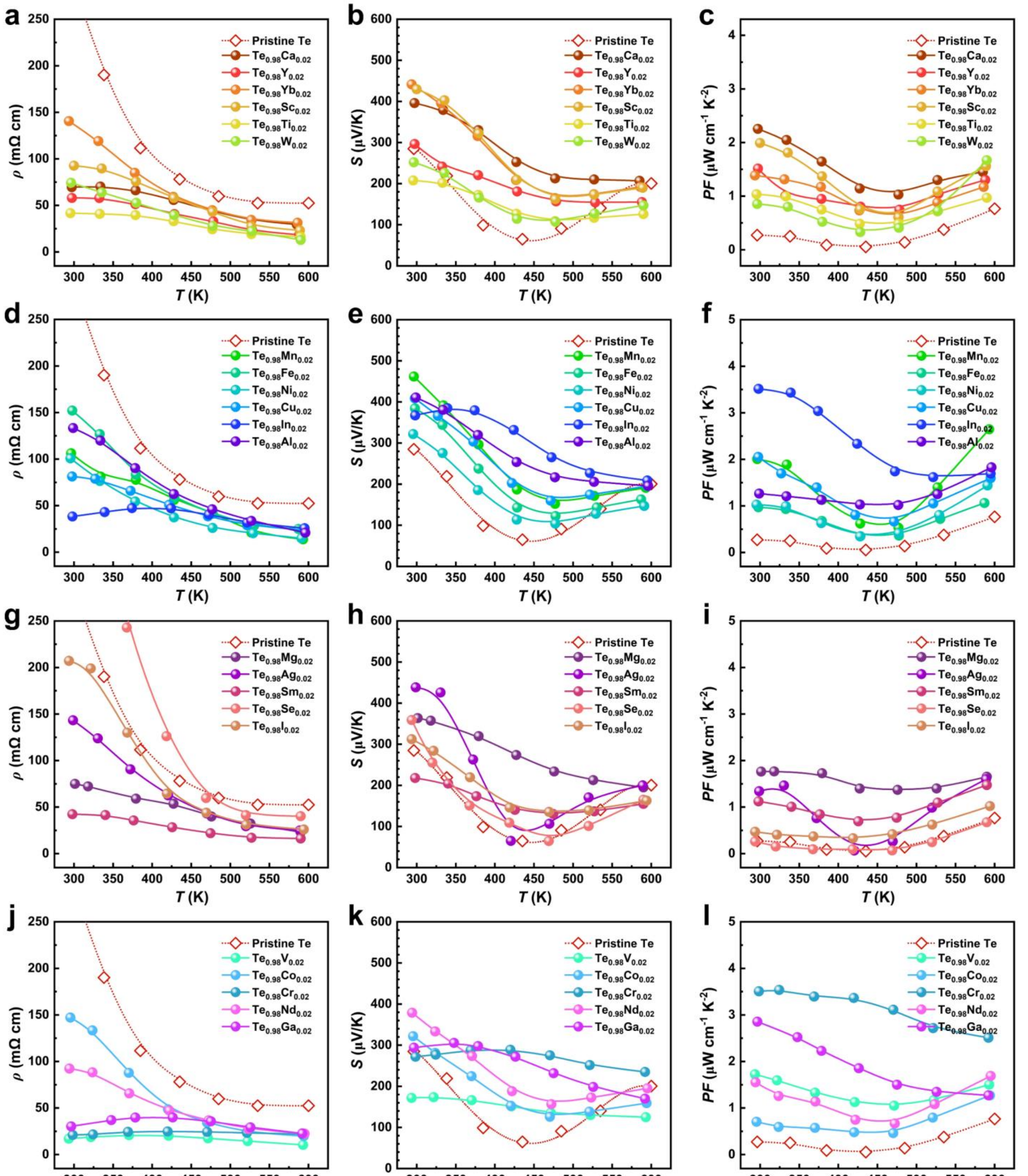

Supplementary Fig. 23. Temperature dependences of electrical properties for 22 different $Te_{0.98}M_{0.02}$ samples and pristine Te. a, b and **c** show the electrical resistivity, the Seebeck coefficient and the PF of samples M=Ca, Y, Yb, Sc, Ti, and W, respectively. **d, e** and **f** show the electrical resistivity, the Seebeck coefficient and the PF of samples M=Mn, Fe, Ni, Cu, In, and Al, respectively. **g, h** and **i** show the electrical resistivity, the Seebeck coefficient and the PF of samples M=Mg, Ag, Sm, Se, and I, respectively. **j, k** and **l**

show the electrical resistivity, the Seebeck coefficient and the PF of samples M=V, Co, Cr, Nd, and Ga, respectively. These “M” elements span from group IA to group VIIA. As expected, doping-induced non-MVB tellurides do not cause a remarkable enhancement of bulk thermoelectric performance here.

References

1. Lin S., Li W., Zhang X., Li J., Chen Z. & Pei Y. Sb induces both doping and precipitation for improving the thermoelectric performance of elemental Te. *Inorg. Chem. Front.* **4**, 1066-1072 (2017).
2. Snyder G. J. & Toberer E. S. Complex thermoelectric materials. *Nat. Mater.* **7**, 105-114 (2008).
3. Vi V. T. T., Nguyen C. Q., Hoi B. D., Phuc H. V., Nguyen C. V. & Hieu N. N. Chemical functionalization of SnAs monolayer: a first-principles study of SnAsX (X = Cl, Br, and I) monolayers. *J. Phys. D: Appl. Phys.* **55**, 505302 (2022).
4. Hasani N., Rajabi-Maram A. & Touski S. B. Strain engineering of electronic and spin properties in SnX (X = P, As, Sb, Bi) monolayers. *J. Phys. Chem. Solids* **174**, 111131 (2023).
5. Han J. C., Wu C. Y., Sun L., Gong H. R. & Gong X. Influence of trigonal deformation on band structure and Seebeck coefficient of tellurium. *J. Phys. Chem. Solids* **135**, 109114 (2019).
6. Peng H., Kioussis N. & Snyder G. J. Elemental tellurium as a chiralp-type thermoelectric material. *Phys. Rev. B* **89**, (2014).
7. Qian X. et al. Effective dopants in p-type elementary Te thermoelectrics. *RSC Adv.* **7**, 17682-17688 (2017).
8. Wu Y., Zhang Q., Liu F., Fang T., Zhu T. & Zhao X. Scattering mechanisms and compositional optimization of high-performance elemental Te as a thermoelectric material. *Adv. Electron. Mater.* **6**, 2000038 (2020).
9. An D. et al. Low thermal conductivity and optimized thermoelectric properties of p-type Te-Sb_2Se_3: synergistic effect of doping and defect engineering. *ACS Appl. Mater. Interfaces* **11**, 27788-27797 (2019).
10. Zhu M. et al. Unique bond breaking in crystalline phase change materials and the quest for metavalent bonding. *Adv. Mater.* **30**, e1706735 (2018).
11. Mishra S. K., Satpathy S. & Jepsen O. Electronic structure and thermoelectric properties of bismuth telluride and bismuth selenide. *J. Phys. Condens. Matter.* **9**, 461 (1997).
12. Vaney J. B. et al. Electronic structure, low-temperature transport and thermodynamic properties of polymorphic β-As_2Te_3. *RSC Adv.* **6**, 52048-52057 (2016).
13. Guarneri L. et al. Metavalent bonding in crystalline solids: how does it collapse? *Adv. Mater.* **33**, 2102356 (2021).
14. Wuttig M., Schön C.-F., Lötfering J., Golub P., Gatti C. & Raty J.-Y. Revisiting the nature of chemical bonding in chalcogenides to explain and design their properties. *Adv. Mater.* **35**, 2208485 (2023).
15. Yu Y. et al. Doping by design: enhanced thermoelectric performance of GeSe alloys through metavalent bonding. *Adv. Mater.* **35**, 2300893 (2023).
16. Yu Y., Cagnoni M., Cojocaru-Mirédin O. & Wuttig M. Chalcogenide thermoelectrics empowered by an unconventional bonding mechanism. *Adv. Funct. Mater.* **30**, 1904862 (2019).
17. Wu R., Yu Y., Jia S., Zhou C., Cojocaru-Miredin O. & Wuttig M. Strong charge carrier scattering at grain boundaries of PbTe caused by the collapse of metavalent bonding. *Nat. Commun.* **14**, 719 (2023).
18. Cheng Y. et al. Understanding the structure and properties of sesqui-chalcogenides (i.e., V_2VI_3 or Pn_2Ch_3 (Pn = Pnictogen, Ch = Chalcogen) compounds) from a bonding perspective. *Adv. Mater.* **31**, 1904316 (2019).
19. Xu Y. et al. Materials screening for disorder-controlled chalcogenide crystals for phase-change memory applications. *Adv. Mater.* **33**, 2006221 (2021).
20. Biswas K. et al. Strained endotaxial nanostructures with high thermoelectric figure of merit. *Nat. Chem.* **3**, 160-166 (2011).
21. Li J., Wang Y., Zhang G., Chen D. & Ren F. First-principles investigation of the electronic structures and Seebeck coefficients of PbTe/SrTe interfaces. *J. Appl. Phys.* **125**, 035107 (2019).
22. Waag A. et al. Molecular-beam epitaxy of beryllium-chalcogenide-based thin films and quantum-well structures. *J. Appl. Phys.* **80**, 792-796 (1996).
23. Yang J.-H., Chen S., Yin W.-J., Gong X. G., Walsh A. & Wei S.-H. Electronic structure and phase stability of MgTe,

ZnTe, CdTe, and their alloys in the B3, B4, and B8 structures. *Phys. Rev. B* **79**, 245202 (2009).

24. Kim J., Choi J. & Kang Y. First-principles study of SrTe and BaTe: promising wide-band-gap semiconductors with ambipolar doping. *Curr. Appl. Phys.* **48**, 90-96 (2023).
25. Zheng Y. et al. Designing hybrid architectures for advanced thermoelectric materials. *Mater. Chem. Front.* **1**, 2457-2473 (2017).
26. Gayner C. & Amouyal Y. Energy filtering of charge carriers: current trends, challenges, and prospects for thermoelectric materials. *Adv. Funct. Mater.* **30**, 1901789 (2020).
